# The Impact of COVID-19 Related Social Distancing on Mental Health Outcomes: A Transdiagnostic Account

**DOI:** 10.3390/ijerph19116596

**Published:** 2022-05-28

**Authors:** Daniella Spencer-Laitt, Elizabeth H. Eustis, David H. Barlow, Todd J. Farchione

**Affiliations:** Center for Anxiety and Related Disorders, Boston University, Boston, MA 02215, USA; eeustis@bu.edu (E.H.E.); dhbarlow@bu.edu (D.H.B.); tfarchio@bu.edu (T.J.F.)

**Keywords:** COVID-19, anxiety, emotional disorder, neuroticism, avoidance, unified protocol

## Abstract

The COVID-19 pandemic, and the social distancing practices that followed, have been associated with increased prevalence of emotional disorders. However, not all individuals affected by COVID-19-related social distancing experienced elevations in emotional disorder symptoms. Understanding this phenomenon is of crucial public health significance given the burden of emotional disorders on individuals and systems. In this narrative review, we consider the differential impact of COVID-19-related social distancing on mental health outcomes from a transdiagnostic perspective. We argue that individuals high in negative affect and aversive reactivity to emotion, that is, neuroticism, and who respond to such emotional experiences with emotion-motivated avoidant coping, are most likely to experience emotional disorders in the context of COVID-19 social distancing. We acknowledge the pro-social and adaptive function of some types of avoidance during the pandemic, which may have initially buffered against negative mental health outcomes. Implications of this conceptualization for treatment of emotional disorders in the present sociocultural context are discussed.

## 1. Introduction

Fourteen-year-old Aya poignantly describes the experience of social distancing during the Coronavirus Disease 2019 (COVID-19) pandemic: “I felt like I was trapped in my own little house, and everyone was far away” [1]. While effective in containing infection, social distancing demanded a dramatic change in lifestyle. This practice has contributed to a multi-dimensional public health crisis: in addition to the high morbidity and mortality rates of COVID-19 itself and incidents of long-term negative health repercussions from even mild forms of the virus (“long COVID” [2]), the worst hit countries are facing widespread increases in mental disorders such as post-traumatic stress disorder, depression, and anxiety; see, e.g., [3,4]. As such, psychological fallout from COVID-19 has been characterized as the “inevitable” next pandemic [5] and has been linked to economic hardship, misinformation, disease-related fears, and social isolation associated with social distancing practices [6].

The connection between increased mental health concerns and COVID-19 social distancing is apparent, though most studies of the mental health consequences of COVID-19 and previous pandemics have focused on the disease itself and not the specific effects of social distancing [7]. Here, we use “social distancing” as an umbrella term to include both stay at home orders by governments as well as other personal decisions people have made to maintain distance to minimize risk of exposure to the virus, given that, at least in the US population, evidence demonstrates minimal differences from mandated compared to self-imposed social distancing on mental health outcomes [8]. Generally, social distancing has been associated with depression, generalized anxiety, acute stress, and intrusive thoughts, e.g., [5,6,7,8]. While this association was hypothesized to be linked to loneliness and lack of social support [9], it has been shown to persist even when level of perceived support is high [8]. Accordingly, while the absence of social support clearly has an impact on a person’s emotional well-being during the pandemic via its effect of increasing loneliness (e.g., [10]), it does not, in our view, entirely explain the psychological consequences of social distancing.

Despite this evidence of elevation in mental health concerns in the context of social distancing, independent of levels of social support, the mechanisms driving this increase have not been clearly elucidated. Moreover, it is unclear why social distancing has had a differential impact: while some people experienced new onset or worsening of emotional disorders, others did not [11]. Having a pre-existing mental health condition is associated with social-distancing-related distress, but this effect is small in magnitude [12]. In addition, there is evidence that those with pre-existing anxiety disorders were less affected by social distancing compared to those with other pre-existing diagnoses such as depression [13]. The goal of this paper, therefore, is to explicate the differential impact of COVID-19-related social distancing on mental health outcomes from a transdiagnostic perspective and to suggest relevant adaptations to treatment approaches.

To explain mental health outcomes, and particularly prevalence of emotional disorders, in the context of COVID-19 social distancing, we adopt the functional definition of emotional disorders, which characterizes such disorders as involving frequent, intense, excessive negative emotions and aversive reactivity to negative emotional experiences [14,15]—that is, neuroticism [16]. We contend that individuals high in neuroticism, and who also display associated emotion-motivated avoidant coping, are, theoretically, most likely to experience emotional disorders following social distancing. However, we note that some types of avoidance may have initially buffered against negative mental health outcomes and may have been adaptive in the context of personal and family risk factors. Only excessive and maladaptive avoidance is associated with emotional disorders in our conception.

It is important to note that the impact of social distancing in the context of COVID-19 might be partially explained by a person’s politics, media consumption, and the beliefs of their social circle as well as demographic and health risk factors. Therefore, for the purposes of our discussion, we divide populations experiencing social distancing into three groups. Group one comprises the proportion of the population who were non-compliant with social distancing measures, perhaps due to the belief that such measures are an unnecessary and excessive response. Group two comprises individuals who have been unable to socially distance due to work or family responsibilities. Group three are those individuals who substantially altered their pattern of social interaction because of the pandemic. Included in group three are those individuals who were placed under differential stress because of COVID-19 due to increased risk of contracting the virus associated with comorbid conditions and demographic factors. Group three members may have been exposed to increased strain because of COVID-19-related impacts on caregivers, families, and children; see, e.g., [17,18]. The focus of this paper is on the prevalence of emotional disorders in group three.

First, we discuss the association between social distancing and emotional disorders. Second, we consider the role of neuroticism in exacerbating COVID-19-related negative affect. However, our focus is on the transdiagnostic mechanism of emotion-motivated avoidant coping that follows and maintains the cycle of negative emotionality. As such, we consider treatment strategies for addressing emotion-motivated avoidant coping in this context. Overall, using a transdiagnostic approach to explain the prevalence of emotional disorders in the context of social distancing has implications for the way we understand vulnerability to emotional disorders during major public health or other crises that demand a change in lifestyle, as well as priorities for treatment of emotional disorders in our present context.

## 2. Social Distancing and the Prevalence of Emotional Disorders

Several factors are associated with the prevalence of emotional disorders in the context of COVID-19. In general, being female [7], young, e.g., [19,20,21], and unemployed [20,22,23], as well as having previous mental health diagnoses, e.g., [20,24,25], confers increased risk. However, pandemic-related stressors such as concern about infection and, relevantly, social distancing have also been associated with anxiety, e.g., [26,27,28]; depression, e.g., [28]; and post-traumatic stress, e.g., [27] on a global level. For example, in China, quarantine was associated with severe state anxiety [28], and in Germany, distress related to contact restriction remained significantly related to depression and anxiety, controlling for demographic variables [20], with more substantial reductions in social contact associated with poorer mental health.

However, this effect was not uniform. In Saudi Arabia, for example, maintaining at least one meter of social distancing was associated with lower stress and anxiety scores [26]. In India, there were no significant differences in stress, anxiety and depression between respondents who were maintaining social distance and those who were not [25]. These varied outcomes may be explained by two factors. First, different countries approached social distancing in different ways. For example, some countries deployed centralized resources and implemented stringent and uniform social distancing strategies (e.g., France, Hong Kong), whereas others did not have a nationwide strategy (e.g., USA) [27]. Second, at least one study, undertaken in the US, has shown that the reason proffered for social distancing plays a role in whether this practice will generate distress. In that study, youth who were motivated to prevent illness by social distancing experienced more anxiety, whereas those who were socially distant due to being told to do so by others experienced more depressive symptoms [29].

That said, despite some variability in methods of social distancing, there is a clear association between symptoms of emotional disorders and social distancing. For example, a multi-center survey study conducted by 35 research organizations in Europe, North Africa, Western Asia, and the Americas demonstrated that COVID-19-related social distancing was associated with a 44.9% increase in scores on the Short Mood and Feelings Questionnaire, a measure of depression [30]. Further, Ebrahimi et al. [31] found, in a sample of over 10,000 respondents, that anxiety and depressive symptom prevalence had doubled compared to before the pandemic, with individuals who adopted social distancing practices having substantially higher rates of these disorders. Finally, initial longitudinal data disclose that deterioration in mental health because of pandemic stressors such as social distancing was not transient and has been sustained even after restrictions have relaxed [32,33,34].

## 3. Neuroticism and Emotional Disorders

### 3.1. Neuroticism and Public Health Crises

Given that it may be posited that social distancing uniquely contributes to the prevalence of emotional disorders beyond demographic variables and other risk factors, we turn to explaining this phenomenon with reference to transdiagnostic principles. Neuroticism has been recognized as an important construct to consider in the context of public health crises [35], as it is associated with significant public health challenges in and of itself. Neuroticism is linked to more substantial use of mental health and primary care services [36], as well as greater chronicity and worse prognosis of emotional disorders [37,38]. In addition, those high in neuroticism are more likely to worry about unfounded medical complaints [39], and seek services accordingly, further causing individual suffering as well as burdening health care systems.

Neuroticism describes an underlying temperamental vulnerability to emotional disorders such as anxiety and depression, which varies individually [16,40,41,42]. It combines heightened negative emotionality with emotional reactivity [16,42] and, in addition to its mental health outcomes including its association with co-occurrence of mental disorders [43], it predicts lower subjective wellbeing [44], reduced positive affect [45], and poorer physical health [46]. Emotional reactivity refers to underlying vulnerabilities, learned through early life experiences, that are expressed as catastrophic interpretations of events and emotional experience itself as uncontrollable, unpredictable, or intolerable [16].

**Figure 1:**
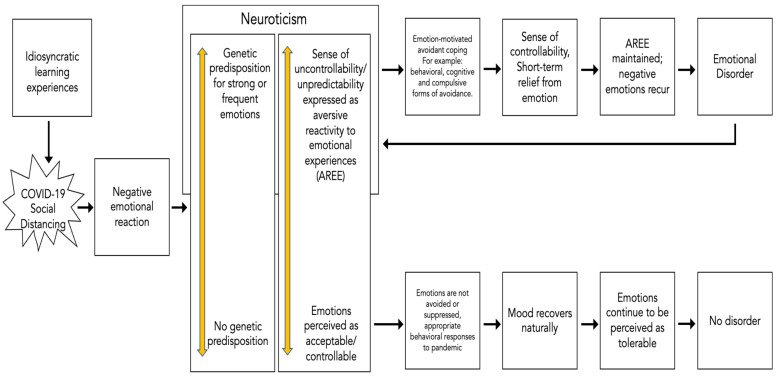
Model of the mechanisms explaining the development of emotional disorders versus adaptive emotional responses (adapted from [16]). COVID-19 social distancing triggers emotional reactions universally. The top pathway indicates that people who have a more neurotic temperament, and who may have developed a sensitivity to social-distancing-related triggers due to idiosyncratic learning experiences, engage in avoidant coping (e.g., rumination, worry, experiential avoidance), leading to the development and maintenance of emotional disorders. The bottom pathway describes an adaptive response to social distancing associated with low neuroticism and non-avoidant coping, leading to no disorder.

Negative emotionality and emotional reactivity are generated when a genetic predisposition to experiencing labile negative affect combines with learned vulnerabilities that are associated with intolerance of those emotions [16]. In a transdiagnostic model of emotional disorders [16], the experience of negative emotions together with a sense of emotions being uncontrollable leads to avoidant cognitive and behavioral coping styles [40]. As we discuss, avoidant coping can lead to a sense of short-term relief but ultimately increases the frequency of negative emotions [16]. As such, Barlow et al. [16] link neuroticism and emotional disorders through avoidant coping (Figure 1).

### 3.2. Neuroticism, Social Distancing, and Mental Health Outcomes

Despite the public health significance of neuroticism, there has been limited research on the construct of neuroticism, according to the definition we adopt, in the context of COVID-19, and none which considers the specific effects of social distancing. Kroencke et al. [47] found that neuroticism, defined as individual differences in negative emotionality, predicted variability in negative affect level during the COVID-19 pandemic in Germany. More specifically, individuals with higher neuroticism also experienced higher mean levels of negative affect in daily life along with more frequent emotional lability (variations in negative affect). Neuroticism predicted negative affect over and above variables such as age, sex, living situation, education or occupation, specific effects of the pandemic itself such as having symptoms of COVID-19, and presence or absence of social interactions [47].

Shokrkon et al. [48] considered individual differences in emotional reactions to COVID-19-related social isolation with reference to neuroticism in a Canadian sample. Of note, the authors conceptualize neuroticism, defined as the tendency to experience negative affect, as a component of the five-factor model of personality [39], which is a somewhat narrower definition than that adopted by this paper [48]. Results demonstrate that despite speculation, and some evidence [49,50] that those higher in trait extraversion would struggle more with pandemic-related social isolation due to the inability to seek energizing interactions with others, it is trait neuroticism that demonstrates significant and negative association with emotional, psychological, and social wellbeing in the context of lockdown measures. Relevantly, there is a high correlation between loneliness, defined as negative responses to the discrepancy between desired and actual social relationships [51], and neuroticism [52,53,54,55,56,57]. Further, individuals higher in neuroticism are more likely to be reactive to social stressors in general [58]. Despite these associations, neuroticism and loneliness are distinct constructs and should not be conflated [52].

Individuals higher in neuroticism tend to react more negatively to stressors [59]. They often have an attentional bias towards negative stimuli, including internal cognitive stressors such as worries, and will experience an increase in the intensity of negative emotional experience when presented with such stimuli (reactivity) [16]. Higher levels of affective reactivity are linked to more experiences of negative affect as well as greater variability in the affective experience [16]. In the context of COVID-19 social distancing, we consider that individuals high in neuroticism are more likely to pay attention to and be preoccupied with the crisis and its negative sequalae, including the requirement to socially distance, and experience negative emotions as a result.

## 4. Aversion, Avoidance, and Social Distancing

We posit that in the context of COVID-19, individuals engaging in social distancing who are high in neuroticism display catastrophic interpretations of COVID-19-related stressors, such as excessive worry about the severity or contagiousness of the virus, or believing isolation and loneliness are permanent (i.e., aversive reactivity). Such aversive reactivity generated by COVID-19 stressors likely leads to the desire to avoid uncomfortable emotional experiences through avoidant cognitive and/or behavioral responses, referred to as emotion-motivated avoidant coping (EMAC). There is evidence for this position: in an international survey of the psychological impact of COVID-19, Passavanti et al. [60] administered the Brief-COPE, a measure designed to examine avoidant- and approach-related coping styles. The highest scores in avoidant coping were associated with high scores on measures of depression and anxiety symptom severity. In another study, Secer et al. [61] found that “experiential avoidance”, avoidance of internal thoughts or feelings [62] which we capture under EMAC, mediated the relationship between fear of COVID-19 and OCD symptoms in a sample of Turkish adolescents.

EMAC can be expressed in behavioral or cognitive strategies aimed at reducing or preventing negative emotion. Here, we consider three such strategies deployed in the context of social distancing that are common features of emotional disorders and are often targeted in treatment [63]. First, rumination and worry about the effects of COVID-19 (cognitive avoidance); second, avoiding places, situations, or other people which may increase perceived risk of COVID-19 (behavioral avoidance); and third, actions such as excessively checking news about COVID-19 or reassurance seeking (checking behaviors).

### 4.1. Cognitive Avoidance

Some level of worry and rumination about the dangerousness of COVID-19 is natural and appropriate in view of associated morbidity and mortality, but when it reaches excess, it can be maladaptive [12]. Rumination largely describes repetitive fixation on negative thoughts about the past, while worry describes repetitive thoughts about the future [64]. This difference is considered relatively minor, given that both worry, and rumination have a shared underlying process of repetitive negative thinking [64]. In the COVID-19 literature, worry and rumination have largely been considered separately.

Rumination has been divided into two sub-types in the context of major crises [65]. While intrusive (or maladaptive) rumination represents involuntary entry of thoughts into awareness followed by excessive pre-occupation with these thoughts, deliberate rumination is a problem-solving approach to making meaning and can lead to post-traumatic growth [65]. Intrusive or maladaptive rumination was positively associated with psychological distress during COVID-19 in a sample of 316 adults in South Korea, a result attributed to the disruption in life associated with COVID-19 [65]. Meanwhile, in the context of social distancing, Hoffart et al. [66] found, in a sample of over 10,000 Norwegian participants, that rumination and worry was associated with higher loneliness scores during COVID-19 social distancing with a medium effect size. This bidirectional effect of rumination and social isolation was also found by Arslan et al. [67], who reported that in college students, loneliness predicted rumination, and was also a mediator in the association between COVID-19 anxiety and rumination. That is, social-distancing-related loneliness was associated with anxiety about COVID-19, and COVID-19 anxiety was positively correlated with rumination.

Excessive worry about COVID-19 has been considered the “core” of what is described as COVID-19 stress syndrome [12]. Taylor [12] found that this factor was the strongest predictor of stress during self-isolation, over and above age or past year mental health conditions, and identified several different types of worry, including worry about infection, worry about socioeconomic fallout and supply chain issues, and worry about others (i.e., xenophobic fears regarding disease spreading). Other studies have also found associations between worry during COVID-19 and mental health outcomes, but have focused in a unidimensional manner on fear of infection, e.g., [68,69]. Such studies have positioned worry in the context of COVID-19 as primarily related to illness anxiety, drawing on research from previous public health crises [70].

Overall, it seems likely that when individuals feel socially isolated due to COVID-19 social distancing practices, they ruminate and/or worry. However, both rumination and worry are theorized to operate functionally to allow individuals to avoid confronting aversive affect and are a common emotion regulation strategy amongst the emotional disorders [16]. Worry and rumination about COVID-19 in individuals high in neuroticism are likely to operate in a circular and mutually reinforcing manner: increased attentional bias towards COVID-19 negative stimuli leads to negative affect, which triggers worry and rumination as an attempt to downregulate the affect. However, this attempt fails, leading to more negative affect, which in turn leads to more attention towards the negative stimuli.

### 4.2. Behavioral Avoidance

From an evolutionary perspective, the emotions of anxiety and fear are designed to elicit behaviors required to effectively respond to and survive threatening situations. In the context of the COVID-19 pandemic, behavioral avoidance, that is, avoidance of places, people or things which increase susceptibility to infection, may be considered adaptive, particularly during lockdown periods or acute spikes in the virus, or throughout the pandemic for those at increased risk of severe illness. Such behavior has public health benefits and demonstrates pro-social compliance with rules and regulations. For example, for some, working from home became the new normal. In addition, people who experience little anxiety about viral outbreaks are less likely to perform hygiene behaviors or get vaccinated [70], indicating that a degree of anxiety and associated behavioral avoidance may be helpful in containing the pandemic’s threats.

It is also important to consider that a proportion of the population had pre-existing patterns of behavioral avoidance, for example, avoidance of social situations as in social anxiety disorder or of reminders of traumatic events as in post-traumatic stress disorder [71]. These pre-existing behavioral patterns may have initially buffered against the negative effects of social isolation since habitual behavior designed to produce a short-term reduction in anxiety was socially sanctioned. This may explain why cross-sectional data do not demonstrate substantially worse mental health outcomes for individuals with pre-existing emotional disorders [12]. However, avoidance of negative emotion in the short term has been demonstrated to increase such negative emotion in the long term, e.g., [72]. Given the widely evidenced relationship between avoidant coping strategies and higher risk of psychopathological symptoms [73,74,75], longitudinal data may very well reveal more significant effects of COVID-19 on the mental health of individuals with pre-existing emotional disorders.

Irrespective of pre-existing mental health conditions, we argue that behavioral avoidance can reach excess when combined with catastrophic beliefs about the pandemic (i.e., aversive reactivity associated with neuroticism). One example of the negative effects of excessive behavioral avoidance is the decline in accessing of care for routine and acute non-COVID-19-related health concerns. Since the outbreak of the COVID-19 pandemic, emergency care has decreased by 23% for heart attacks, 20% for strokes, and 60% for ambulatory visits [76], increasing risk for both short- and long-term negative health outcomes. In a study of US adults, symptoms of depression and anxiety were strongly correlated with avoidance of medical care during the pandemic [76]. This may be explained by both purposeful avoidance of emergency rooms and medical centers to reduce the chance of infection, and avoidance of relevant health-related information [77]. Behavioral avoidance also decreases access to healthy food, exercise, and social interaction, all of which contribute to psychological wellbeing [78]. Moreover, as noted above, such behavior is designed, and is often successful in, reducing short-term distress. In the long term, however, it is related to higher risk of symptoms of anxiety and depression [73], poorer health and psychological wellbeing [76], and can interfere with achievement of individual goals.

### 4.3. Safety and Checking Behaviors

A third category of avoidance is what has been described both as checking and safety behaviors [79] and compulsive checking [12]. Such behaviors can include seeking reassurance about the pandemic or its risks, excessively checking news reports, or performing hygiene or cleaning routines, activities which provide one with an “illusion of control”, that is, a belief one has greater control over events than is the case. Some of these behaviors (e.g., hand washing) were recommended by public health authorities, while others (e.g., news checking) were not and instead were primarily driven by pre-occupation with the crisis [80]. Like the behavioral avoidance described above, such behaviors may be adaptive to an extent: they provide a temporary sense of control over the feeling of uncertainty surrounding how the virus is spreading, increasing numbers of cases, and the realistic risk of contact with the virus and may be necessary to contain infection. However, such behaviors can be maladaptive when performed excessively, that is, these rituals take up significant amounts of time, cause high levels of distress, and interfere with functioning.

In a study of US undergraduates, Knowles et al. [80] found that there was a strong correlation between COVID-19 anxiety and COVID-19-related “safety behaviors”, including reassurance seeking, cleaning rituals, and news checking. In Greece, safety and checking behaviors were associated with higher levels of COVID-19-related fear [79]. Compulsive behaviors, including safety and checking behaviors, are considered to maintain anxiety and fear because they generate an increase in the perceived importance of the threat [81] and prevent unlearning of the association between excessive rituals and COVID-19 infection. Individuals pre-occupied with the COVID-19 crisis are more likely to perform safety and checking behaviors, and then may be driven to perform these behaviors at increasing frequency when they do not in fact contract the virus. This may ultimately increase fear and anxiety surrounding contamination, even when the threat has passed or diminished [80].

## 5. Implications for Treatment

Having explained the prevalence of emotional disorders following COVID-19 social distancing as a product of underlying avoidant processes associated with high neuroticism, we now turn to the implications of this model for treatment. Given the shared mechanisms of emotional disorders in general and during the pandemic, adapted components of transdiagnostic, emotion-focused, cognitive-behavioral treatments such as the Unified Protocol for Transdiagnostic Treatment of Emotional Disorders (UP) [63,82] may be well suited for responding to the expanded need for psychological services given their focus on higher-order factors as opposed to specific diagnoses. The UP is an emotion-focused treatment geared toward reducing symptoms of emotional disorders that are characterized by the core elements of the model described above: strong negative emotions, aversive perceptions of emotions rooted in maladaptive beliefs about their uncontrollability or intolerability, and avoidant coping aimed at reducing or preventing emotional discomfort. While we do not describe all modules of the UP here or suggest that it is the only viable option for treatment, we highlight specific components of this treatment which may be particularly suited to meeting the challenges of emotional disorders, including complex cases with multiple comorbidities, related to COVID-19 social distancing. We provide a summary of these components and their application to emotional disorders in the context of COVID-19 in Table 1.

### 5.1. Mindful Emotion Awareness

Following psychoeducation on the nature and function of emotions, including why humans have emotions, mindful emotion awareness is introduced in the UP as a skill to help individuals respond to their emotions in ways that are helpful to them. Specifically, instead of judging emotions as negative and rigidly attempting to avoid them, which tends to backfire and increase distress, individuals are encouraged to practice present-focused and non-judgmental awareness of emotional experience. For example, someone may notice that they are feeling anxious about COVID-19, worrying about whether things will ever get better in the future, and avoid safe social activities as a result. Instead of judging themselves and their emotion (e.g., “What’s wrong with me?”, “I shouldn’t be feeling this way.”) and engaging in avoidant behaviors (e.g., maladaptive social isolation), this individual could notice that their thoughts are future-oriented and gently bring themselves back to the present. In addition, they could notice that they are judging their emotions and remind themselves that it makes sense to feel anxious at times during a pandemic. Responding in this way will likely make the emotion feel more tolerable and decrease the need to engage in subsequent behaviors to try to avoid the intense negative affect. Therefore, this module and skill helps to reduce EMAC, in line with our first contention. In addition, this practice, called mindful emotion awareness in the UP, helps to facilitate the use of other cognitive and behavioral skills later in the protocol since individuals who are willing to experience their emotions non-judgmentally may be better able to think flexibly and engage in exposures to emotional experiences.

### 5.2. Cognitive Flexibility

An important component of treatment in the context of emotional disorders associated with COVID-19 social distancing is addressing automatic thoughts about the pandemic such as overestimating the probability that an event will occur or imagining catastrophic outcomes. This process can also help with previously described cognitive avoidance strategies such as pathological rumination or worry. Part of this process includes acknowledging that our context impacts our thoughts and some of these thoughts may be more or less helpful depending on context. Cognitive flexibility in the UP promotes considering alternative appraisals of automatic thoughts. In the context of COVID-19 and the inherent uncertainty about its course, the goal of cognitive flexibility should be to tolerate uncertainty and consider multiple different possibilities for the disease course should COVID-19 be contracted. For example, a person might be encouraged to examine possible outcomes if they contracted the virus: one is certainly serious illness, and another is mild infection, and to evaluate the probabilities of these different outcomes. Further, a person might be encouraged to think about how they would cope with contracting COVID-19 and what specific steps they might take, with the goal of validating that contracting the virus would be difficult while prompting reflection that coping strategies are available for most situations outside of death. Alternatively, a person having the automatic thought that changes to our way of life through wearing masks and maintaining distance are permanent and intolerable might be prompted to draw on previous examples of coping with difficult circumstances to bolster belief in the possibility that the pandemic’s unique burdens can indeed be coped with (e.g., this is a really challenging situation, and I have gotten through hard things before). Overall, the goal of cognitive flexibility in this context is to bolster tolerance of uncertainty, in line with our second contention.

### 5.3. Alternative Action

The types of avoidance described in this paper may be characterized as emotional behaviors, that is, they are actions intended to avoid or reduce emotions and/or can be driven by the emotion itself (e.g., fear drives flight or fight). These behaviors may be adaptive depending on the individual’s context. However, in many situations, these behaviors are often ineffective and non-adaptive. As previously described, such behaviors must be interpreted in our present context: there is a pro-social and protective function to social distancing, and such behavior may be adaptive and protective. It may also provide relief from strong negative emotions associated with the pandemic. However, in the long term, excessive social distancing (for example, avoiding required doctor’s visits or missing important events) interferes with individual goals and well-being. In the UP, individuals identify emotional behaviors they engage in (e.g., avoiding specific places, cognitive avoidance, substance use, worry, etc.) and then identify alternative actions they could engage with instead that are consistent with their goals and allow themselves to experience negative emotion rather than suppressing or attempting to control such emotion.

Some examples of this approach are as follows: for cognitive avoidance such as worry and rumination, a person might be encouraged to maintain mindful emotion awareness. For behavioral avoidance such as excessive social distancing, a person might be encouraged to attend the feared doctor’s visit anyway. For behaviors such as excessive cleaning, a person may be prompted to follow government guidelines. Alternatively, in the case of news consumption, a person might be prompted to consume a “just right” amount of news, for example by setting time limits on news access, that facilitates appropriate engagement in safety behaviors [83]. These alternative actions serve the additional purpose of functioning as behavioral activation. In the service of increasing positive affect, which plays an important role in mood, a further task of treatment could be planning graded assignments broken down into achievable units [84] such as increasing exercise or engaging in appropriate and enjoyable social activities. Overall, alternative action promotes decreased EMAC and increased behavioral activation, in line with our first contention.

### 5.4. Emotion Exposure

In addition to countering the cognitive and behavioral responses to negative emotions, through cognitive flexibility and alternative action, the UP includes a focus on exposure to intense emotional experiences Emotion exposures are situations or activities that are intended to provoke strong emotions. These exposures are personalized to the individual, their goals, and the ways in which they avoid their emotions. The goal of such exposure is to approach and fully experience emotions to facilitate inhibitory learning and extinguish anxiety and distress related to emotions [85]. Through these experiences, individuals learn that emotions are not dangerous and are temporary and manageable [15,86]. In this context, it is important to consider that strong emotions associated with the pandemic, such as anxiety about infection, are appropriate where they serve to facilitate compliance with public health measures and increase personal safety. Accordingly, the goal of emotion exposure is to manage, rather than eliminate, strong emotions so that the emotion is, on the one hand, perceived as manageable, but on the other, continues to prompt adaptive action [86]. The alternative actions described above may serve as emotion exposures when combined with strategies to ensure continued contact with the aversive emotion, such as mindful emotion awareness. In addition, individuals may be prompted to engage in imaginal exposures, for example considering what it would be like if life does not return to normality for some time, or ever. Overall, emotion exposure serves the purpose of distinguishing between maladaptive and adaptive avoidance and reducing the former, in line with our first contention.

We suggest that transdiagnostic interventions, such as the UP, may be appropriate for the treatment of the underlying common processes we have identified as consistent with the differential impact of COVID-19 social distancing on mental wellbeing. Some components of the UP, which are also included in other cognitive behavioral approaches, are particularly useful in targeting sustained avoidance. Specifically, tools of mindful emotion awareness, cognitive flexibility, alternative action, and emotion exposure, serve to increase a sense of negative emotion tolerability while having the added benefit of increasing behavioral activation.

## 6. Conclusions

It is evident that, in the context of the COVID-19 pandemic, there is an increased need for mental health services, as people have invariably been exposed to either the virus itself or loss, financial strain, and social isolation. This paper sets out to explain the prevalence of mental health concerns in the context of COVID-19-related social distancing, and the extent to which social distancing has had differential psychological impacts. We describe an underlying temperamental vulnerability, neuroticism, which predisposes individuals to increased negative affect and greater reactivity to negative stimuli, and typically leads to attempts to avoid emotions. While many individuals experienced negative affect in the context of COVID-19, and some individuals were disproportionately affected by the pandemic and its associated social distancing, we argue that individuals high in neuroticism are more likely to catastrophize COVID-19 stressors and seek to avoid aversive emotional experiences. This avoidance may take several forms: for example, worry or rumination or checking behaviors such as reassurance seeking. We also acknowledged that while avoidance of situations posing COVID-19 risk is adaptive for some individuals, depending on context, it can also contribute to the development of emotional disorders in the long term. We then discussed various targets for treatment in the context of COVID-19 and ways that excessive maladaptive avoidance may be addressed using cognitive-behavioral techniques.

We focused on generating an explanatory framework and providing directions for treatment which are applicable during the current circumstances and future global events that demand a change in individual behaviors and routines. However, the construct of neuroticism, as we describe it, has been subject to very limited empirical research in the context of COVID-19, and is therefore an area for future investigation. Further, as we note throughout, it can currently be difficult to distinguish adaptive and maladaptive avoidance. In a particularly unpredictable time, it is important to contextualize avoidant behaviors within national and global circumstances. As more data on the longer-term impacts of social distancing become available, we encourage a research agenda which focuses on understanding the cumulative effects of pandemic-related stressors, including those associated with social distancing, both in individuals with pre-existing mental health conditions and without.

## Figures and Tables

**Table 1:** Reducing emotion-motivated avoidant coping: techniques from the Unified Protocol.

Skill	Target Behavior/Experience	Example Application
Mindful Emotion Awareness **Goal:** Practice present moment, nonjudgmental awareness in response to emotions	Future- and past-focused awareness such as pre-occupation with life before COVID-19 or the prospect that the world will not return to normality in the future	Practice bringing awareness back to the present moment, again and again
	Aversive reactions or judgments about emotions such as judging negative affect experienced during COVID-19 as “bad” and “intolerable”, or judging oneself as “bad” or “weak” for experiencing emotions	Practice being non-judgmental of emotions in response to judgments
	Attempts to avoid emotions for example through distraction, cognitive or behavioral avoidance, or checking behaviors	Practice being willing to experience emotions vs. avoid them
Cognitive Flexibility **Goal:** Increase tolerance of uncertainty	Rumination about COVID-19 stressors Worry about COVID-19 stressors and associated catastrophic fears	Consider the multiple different outcomes possible if COVID-19 is contracted Consider previous times when the individual has coped with uncertainty
Alternative Action **Goal:** Exposure to negative emotions, reduce non-adaptive behaviors, increase behavioral activation	Excessive avoidance of necessary activities that interferes with functioning (e.g., doctor’s visits)	Attend doctor’s visits
	Compulsive checking of news	Limit news checking to certain times of day for time-limited periods
	Compulsive cleaning Reassurance seeking	Follow guidelines from authorities Rely on self for reassurance
Emotion Exposure **Goal:** Perceive negative emotions as manageable, retain adaptive levels of anxiety and other emotions	Attempts to avoid or control strong negative emotions associated with COVID-19 social distancing such as anxiety	Mindful emotion awareness Imaginal exposure of worse case scenarios (contracting COVID-19, unlimited restrictions to our way of life) Attending a doctor’s appointment Plan a social interaction (e.g., get together in-person inside, get together outside, via Zoom)

## Data Availability

Not applicable.
